# Do Clowns Really Taste Funny? An Investigation of the Relationship between Humor and Playfulness in Clown Doctors

**DOI:** 10.3390/bs13040328

**Published:** 2023-04-12

**Authors:** Alberto Dionigi, Alessandra Fermani, Carla Canestrari

**Affiliations:** 1 Studio Psi.Co., 47841 Cattolica, Italy; 2 Department of Education, Cultural Heritage and Tourism, University of Macerata, 62100 Macerata, Italy; alessandra.fermani@unimc.it (A.F.); carla.canestrari@unimc.it (C.C.)

**Keywords:** humor, playfulness, clown doctor, comic styles, healthcare clowning

## Abstract

Healthcare clowning represents a well-established method for relieving patients and their relatives of discomfort during hospitalization. Although studies concerning the effectiveness of this approach are increasing in number, state-of-the-art studies conducted to evaluate the psychological characteristics of clown doctors are scarce. In this cross-sectional study, a convenient sample of 210 clown doctors (143 females, 67 males) aged between 18 and 75 years (M = 47.34, SD = 12.31) completed a demographic questionnaire, the Comic Styles Markers, and the Short Measure for Adult Playfulness. The results demonstrated that clown doctors bring higher levels of fun, benevolent humor, and nonsense and a lower level of cynicism compared to the populace. Moreover, the participants with more experience tend to use less irony, sarcasm, and cynicism than those with less experience. Playfulness was primarily related to the lighter styles of humor, and specific differences between the Whiteface and the Auguste clown doctors were observed. The results are discussed with reference to previous studies conducted on groups of clown doctors.

## 1. Introduction

In the last 20 years, interest in understanding the role and function of clown intervention in healthcare settings has been growing. The clown, a comic character that entertains people, has recently entered the healthcare setting owing to its empathic and humorous functions. Nowadays, healthcare clowning represents a well-documented complementary and alternative medicine approach aimed at providing a humor-based distraction to improve patients’ moods and the quality of their stay [1]. Healthcare clowning was first practiced in 1986 in New York City (USA) by Michael Christensen—a professional clown who began working in hospitals under a program called the Big Apple Circus Clown Care Unit. This new figure was called a “clown doctor”, as it parodied the work of medical doctors by donning a colorful doctor’s coat over the clown costume to induce positive emotions in patients, their relatives, and healthcare staff. These practitioners are organized into clown care units (CCUs) that comprise humor practitioners who either work alone or in pairs. Clown doctors play a different role than what their colleagues in the circus perform as, besides being skilled clowns, clown doctors must be specifically trained in psychological and relational expertise because of the sensitive environment they work in [2]. They should be able to uplift the patients’ mood by delivering something positive and unconventional while focusing on the recipients’ psychological conditions and the impact that their intervention can exert. Healthcare clowning is, therefore, aimed at promoting the psychological well-being of patients, thereby improving their overall health and relieving them of the fear, stress, and anxiety associated with their treatment and being in an unfamiliar environment [3,4,5]. Moreover, the intervention is also aimed at promoting imagination, creativity, talent, and communication among patients [6,7].

Clown doctors usually work in pairs, playing two primary characters: the Whiteface and the Auguste. The Whiteface clown is elegant, serious, repressive, and authoritarian, representing the concepts of structure, order, rules, and preparation. Conversely, the Auguste clown is clumsy, goofy, and more ridiculous, encapsulating notions of spontaneity, disruption, impulsivity, and playfulness. The comic effect of a clown gag is generated by the contrast between these two figures [8].

At a state-of-the-art level, healthcare clowning encompasses a large variety of practitioners, including both professional and volunteer clown doctors who undergo different types of training [9]. Although several studies have investigated the role of clown intervention in improving patients’ moods and decreasing negative emotions, only a few have evaluated the psychological and artistic characteristics of this new character. Moreover, quantitative research in this field is scarce and limited to specific cultures. An early study on this topic demonstrated that clowns possess specific personality traits compared to the general population: clown doctors are more extroverted, conscientious, open to experiences, agreeable, and less neurotic [10]. Another empirical study was conducted on the psychological aspects of clowns to better understand the essence of being a clown doctor [11]. This research evaluated the core aspects of personality that can facilitate or inhibit the “clown shift”, that is, the transition between the person and the clown character and vice versa. The study’s core finding was that being a clown leads to a person assuming a cognitive state of mind that allows them to perceive the world and act in a particular manner where everything (or almost everything) is permitted. This transition occurs when the person switches from a habitual state of mind to a clown state of mind and vice versa. The study results highlighted that positive beliefs and reflective awareness facilitate the clown shift, while cognitive interferences and anxiety hinder the process. Moreover, neuroticism, which was found to be strictly correlated with pre-activity anxiety and worry during practice, emerged as a negative factor for this transition [11]. Further studies have shed light on the empathetic skills required to serve as clowns. Theoretically, clown doctors are required to possess high empathy to glean and respond to the emotional state of patients. Empirical findings have demonstrated that empathy is a key factor contributing to psychological health and over and above self-efficacy and optimism, confirming that empathy constitutes an important skill for clown doctors [12]. Finally, a recent study [13] indicated that clown doctors showed the lowest fear of being laughed at (gelotophobia) and used higher levels of humor as a coping strategy compared to people who attended healthcare training courses related to humor and to people who attended healthcare training courses not related to humor (control group). Moreover, clown doctors showed a greater use of the affiliative, self-enhancing, and aggressive humor styles, particularly in comparison to the control group.

The clown is a comic character; therefore, we can assume that these practitioners should possess a high sense of humor. However, research on the evaluation of clown doctors’ sense of humor is relatively scarce, limited to a few qualitative studies conducted on small samples. One of the first qualitative studies aimed at identifying the peculiarities of clowns’ activities found artistic and psychological potential in working in pairs. Specifically, clowns’ strategy of working in pairs enhances their ability to treat patients with empathy. Another aspect that emerged concerned humoristic communication, which plays a critical role in the relationship between a clown doctor and a patient by allowing an open space for play together [14]. A further study was conducted over a period of seven years to evaluate which psychological characteristics are essential for clown doctors to perform their activities [15]. The findings revealed that clown doctors could provide a quality of care that goes beyond the boundaries, creating a safe space for hospitalized children where they can be offered relief and the opportunity to see the lighter side of their condition. The prerequisites to achieve this result are cheerfulness, laughter, and humor—all of which add an element of surprise that instills a positive attitude to face a negative condition. Another qualitative study was conducted in Israel to achieve a better understating of clowns’ emotions regarding their work with adults suffering from chronic diseases [16]. Again, humor constituted a fundamental skill for addressing these difficult situations and employing an adaptive coping strategy to deal with the stress. Moreover, a recent qualitative study conducted on a sample of therapeutic clowns working in Canada reported that both playfulness and creativity are two fundamental skills required to work both in person and online [7].

Humor can be seen as a special variant of play; therefore, playfulness represents the basis for a sense of humor [17], with a special focus on the fun and amusement component [18]. Numerous studies have shown how playfulness helps relieve stress, improves brain function, boosts creativity, improves connections with others, and heals emotional wounds [19]. This is consistent with the role and function of clown doctors, which is aimed at improving patients and their relative mood during hospitalization.

Although some qualitative studies have highlighted these characteristics required in clown doctors to perform their activity (i.e., humor and playfulness), to date, no empirical study has been conducted to evaluate these variables. Therefore, this study aimed to evaluate humor and playfulness in a sample population of Italian clown doctors and investigate whether they are related to specific characteristics of clown doctors themselves, such as their age, the role they played during the activity, and their experience. Sense of humor has often been assumed to be positive, fulfilling, and healthy [20]; however, several studies have indicated that humor also has a dark and unhealthy side to it [21]. In this regard, recently, a new model of humor focused on a list of eight lower-level comic styles has emerged, namely the Comic Style Markers (CSM) [21]. These eight comic styles that can be differentiated into lighter or darker styles of humor constitute Fun, Humor, Nonsense, Wit, Irony, Satire, Sarcasm, and Cynicism [22]. The four lighter styles, which relate to benign and social effects, behaviors, cognitions, and goals, are: (1) Fun, aimed at spreading good mood and good companionship; (2) Humor, aimed at adopting a benevolent view about the faults of people; (3) Nonsense, based on playing with incongruities and ridiculousness with no specific purpose; and (4) Wit, related to the ability to play with ideas and thoughts, thereby creating clever connections. On the other hand, darker styles are primarily based on mockery and ridicule. They are: (1) Irony, reflecting a contrast or incongruity between expectations of a situation and reality and indicating the opposite of what is meant; (2) Satire, wherein humor is employed to criticize and correct shortcomings, misconduct, and moral wrongdoings; (3) Sarcasm, based on being critical of others to mock someone or something; and (4) Cynicism, exhibiting a negative and destructive attitude aimed at devaluing commonly recognized values. Therefore, we investigated specific forms of humor for a better understanding of the role and functions of the clown activity.

At the time of writing this paper, no study had investigated playfulness and the use of specific types of humor by clown doctors. The first aim of this study was to compare the differences between the two clown characters, the Whiteface and the Auguste, in how they utilize the eight comic styles. We expected the Auguste to mainly relate to the lighter styles and the Whiteface to the darker styles. The second aim of the study was to evaluate whether people wearing the clothes of a clown doctor possess a higher ability to use comic styles compared to the general population. We assumed that clown doctors would rate higher in the lighter styles and lower in the darker ones compared to the general populace. Third, we were interested in investigating the relationship between playfulness and humor, expecting a significant correlation with the lighter styles. Finally, we conducted an explorative investigation on how playfulness and humor would relate to the age, character, and experience of clown doctors. We believe that the findings will contribute to the knowledge in the field and be of great interest for trainers and clown doctors themselves. During their training, they can focus on practicing specific categories of humor depending on the character played and therefore, be more effective during their activity in the healthcare setting.

## 2. Materials and Methods

### 2.1. Participants

The total sample comprised 210 clown doctors (143 females, 68.1%; 67 males, 31.9%) aged between 18 and 75 years (M = 47.34, SD = 12.31). All participants were well-educated adults (0.5% primary school; 8.6% low secondary school; 52.4% upper secondary school; 28.1% university; 7.6% master/post degree; and 2.9% doctorate). With regard to marital status, 30.5% were not married, 57.6% were married or cohabiting, 9.5% were divorced, and 2.4% of the participants were widowed. The participants had varying levels of experience in the art of clowning in health settings (M = 7.16 years, SD = 5.32, range = 0–22 years). Based on their experience, we separated the clown doctors into three subgroups (8 missing): “<1–4 years” (74; 36.6%); “4–9 years” (63; 31.2%); and “>9 years” (65; 32.2%). The majority of the participants (N = 195; 92.9%) were volunteers. Furthermore, 122 (58.1%) played the role of the Auguste clown, while 88 (41.9%) played the role of the Whiteface clown.

### 2.2. Instruments

The participants anonymously completed a short demographic questionnaire where they were asked to indicate their age, gender, level of education, and marital status. The questionnaire also included questions related to the clown doctors’ activity: the clowns were asked to indicate which character they mainly played (the Whiteface or the Auguste) and for how long they had been involved in this activity.

The CSM [22] is a self-report questionnaire comprising 48 marker items that capture the eight comic styles of Fun, Humor, Nonsense, Wit, Irony, Satire, Sarcasm, and Cynicism. Six marker items represent each comic style, utilizing a seven-point response format ranging from 1 (strongly disagree) to 7 (strongly agree). Total scores correspond to the mean of the six items, with higher scores corresponding to higher use of that specific comic style. This study employed the Italian version, which was translated and validated by Dionigi et al. [23]. The eight scales showed good to acceptable reliabilities (McDonald’s ω total: Fun = 0.84; Humor = 0.79; Nonsense = 0.83; Wit = 0.82; Irony = 0.76; Satire = 0.78; Sarcasm = 0.73; Cynicism = 0.82).

The Short Measure of Adult Playfulness (SMAP) [24] is a five-item questionnaire for assessing playfulness in adults. It was developed for providing a global, cognitive self-description of playfulness. In this questionnaire, items are rated using a 7-point scale (1 = “strongly disagree”; 7 = “strongly agree”). Greater scores in the SMAP indicate an easy onset and high intensity of playful experiences, along with a frequent display of playful activities. The McDonald’s coefficient for this study was 0.82.

### 2.3. Procedure

The research design was cross-sectional and the data collection was performed online. The inclusion criteria were age ≥ 18 years and being a clown doctor and an Italian citizen. E-mails were sent to the different CCUs, asking affiliated clowns to take the battery of tests. The e-mails contained a link to a survey created on Survio.com and the participants’ anonymity was guaranteed. A total of 253 participants responded to the survey, while the final sample that participated in the study comprised 210 clowns who had fully completed the battery of tests. The survey also contained an explanation of the aim of the study and an agreement of participation. The study was performed in accordance with the local ethics guidelines and was approved by the ethics research committee of the University of Macerata. Furthermore, all the participants were guaranteed anonymity. In order to compare the differences between our sample of clown doctors and the general population, we asked the authors of the study on the Italian validation of the Comic Styles Markers to share their dataset so that we could compare the two results [23]. The study was based on a sample of 607 participants (393 females, 64.7% and 214 females, 35.3%) aged 17 to 77 years (M = 36.50, SD = 13.28).

### 2.4. Data Analysis

Descriptive statistics, multivariate analysis of variance (MANOVA), bivariate correlations, and linear regressions were computed. All statistical analyses were performed using the SPSS v.21.0 statistic software package (IBM Corp., Armonk, NY, USA). Values of *p* < 0.05 were considered statistically significant.

## 3. Results

The present study aimed to examine whether the participants differed in terms of individual levels of playfulness, the eight CSM (Fun, Humor, Nonsense, Wit, Irony, Satire, Sarcasm, and Cynicism) as dependent variables, the three levels of experience (i.e., <1–4 years; 4–9 years; >9 years), and the two characters (the Auguste and the Whiteface) that were independent variables. The analyses showed no statistically significant differences with respect to gender and age. Playfulness had very high scores in all groups (i.e., the three levels of experience and the two characters); however, no statistical significance was found. Instead, the results of the MANOVA analyses (see Table 1) revealed significant statistical differences for Irony, Sarcasm, and Cynicism in the Tukey test in the six groups (two characters and three levels of experience).

The participants showed high levels in all adaptive styles (higher scores than the midpoint of the Likert scale: 4) and low scores in two out of the four darker styles (i.e., Sarcasm and Cynicism). Specifically, those with higher experience (>9 years) were found to employ less irony, sarcasm, and cynicism than those with less experience (<9 years), as evidenced by the Tukey test.

Regarding the role played, according to Wilks’ Lambda criterion, the Whiteface clown doctors used more sarcasm and cynicism than their Auguste counterparts. The results indicated that in combination, the above dependent variables were significantly affected by the characters: Sarcasm (F [1, 210] = 4.34, *p* < 0.05, η^2^ = 0.02) and Cynicism (F [1, 210] = 4.48, *p* < 0.05, η^2^ = 0.02), but not by the interactions between experience and role.

In the second step, we examined whether our sample of clown doctors and people without experience in this area (as independent variables) differed in terms of individual levels in the eight CSM (Fun, Humor, Nonsense, Wit, Irony, Satire, Sarcasm, and Cynicism) as the dependent variables. We compared our dataset with the data obtained by the Italian validation of the CSM [22]. The results of the MANOVA analyses (see Table 2) revealed significant statistical differences. Clown doctors showed higher scores in lighter styles and lower scores in darker styles compared to non-clowns; however, significant statistical differences were found only in Fun, Humor, Nonsense, and Cynicism.

### Correlations and Regressions

To further examine the relationship between playfulness and the eight comic styles, we performed statistical correlations and linear regression analyses on the whole sample and the two characters: the Whiteface and the Auguste (see Table 3). The correlations in the whole sample revealed that Fun, Humor, Nonsense, and Wit were positively associated with playfulness. When considering the two characters, Fun, Humor, Nonsense, and Wit were found to be positively associated with playfulness for both the Whiteface and the Auguste clowns. Moreover, the Auguste clowns showed a positive correlation between Satire and playfulness.

The linear regression findings reported in Table 3 indicated a medium *R* square effect, and the results were noteworthy in general. In the total sample, Fun, Humor, Nonsense, and Wit predicted playfulness positively, while Cynicism was negatively related to playfulness. The Whiteface sample showed that the best predictors of playfulness were Fun and Humor. Finally, in participants playing the role of the Auguste clown, the best predictor of playfulness was Fun with no other significant results.

## 4. Discussion

In recent years, studies conducted to understand the psychological features of clown doctors have been on the rise. However, to date, no study has been conducted to evaluate humor and playfulness in these practitioners. The primary aim of this study was to fill this gap in the literature. In this study, we performed a cross-sectional examination of how specific categories of humor, explicitly the comic styles, and playfulness were related to each other and to the training, level of experience, and roles played in a sample of Italian clown doctors. Overall, the findings supported our hypotheses that both comic styles and playfulness are differently related to the level of experience and the role played by the clowns.

The MANOVA analyses revealed that the participants with more experience (i.e., > 9 years) tended to use less irony, sarcasm, and cynicism than those with less experience (i.e., < 9 years). This result may reflect the tendency of less experienced clowns to employ more maladaptive styles of humor. In particular, the more experienced the clown doctors were, the less ironic and maladaptive humor they used. Due to their expertise, clowns with more experience may have learned to use more adaptive forms of humor, which enabled them to adapt their humorous comments to the peculiar setting they were working in. New or intermediate clowns on the other hand were more prone to utilize darker styles.

In line with our expectations, the Whiteface clowns used more sarcasm and cynicism than the Auguste clowns. This finding reflects the idea that in the duo, the character of the Whiteface clown represents the rational voice of reason and is the orderly decision-maker, being strict, authoritarian, severe, and precise. These traits have been found to relate to both sarcasm and cynicism [25]. People who act as Whiteface clowns may use these specific forms of humor to make light of the situation and their colleagues who embody the role of the Auguste clown, creating comic conflict, the basis for the amusement they offer [8].

In general, the participants exhibited higher levels of adaptive styles as opposed to maladaptive styles, with Sarcasm and Cynicism showing the lowest scores. This result is consistent with previous findings that report how clown doctors are required to use positive forms of humor and gentle play [7,8,26].

When considering the differences in comic styles between the sample of clown doctors and the general population [23], the results showed that the clowns reported significantly higher scores in Fun, Benevolent Humor, and Nonsense and lower in Cynicism compared to non-clowns. This result is in line with the role clowns play in healthcare settings: they are required to use affiliative and positive forms of humor (fun) to amuse the audience [9,27]. Moreover, one of the primary purposes of clown doctors is to address difficult situations and in such settings, humor may serve as a useful coping strategy [9,28]. In this regard, recent studies have demonstrated how benevolent humor may be compared to self-enhancing forms of humor and how fun reflects the ability to use humor in an affiliative way [29]. Fun and Benevolent Humor are the comic styles dedicated to capturing the essence of adaptive humor and are aimed at spreading a good mood and treating everyday struggles with humor and benevolence. Therefore, we would expect a clown dealing with stressful situations to employ these types of humor, as they serve as a means of dealing with stress and promoting well-being [25].

The comic style of Nonsense is based on playing with incongruities and ridiculousness without any purpose and is primarily based on incongruities that are only partly resolved [30]. This type of humor is generally more appreciated by young children, while both appreciation and comprehension of other types of humor increase with the development of cognitive abilities [30,31]. Nowadays, clown doctors are active with patients from all age groups; however, the majority of their work revolves around young children. We can assume that clown doctors have improved the nonsense comic style to be perceived as funnier and to induce a positive mood in their audience. Finally, the lower score regarding Cynicism is not surprising, considering this form of humor is primarily related to exhibiting a negative and destructive attitude aimed at devaluing commonly recognized values [22]; therefore, clown doctors avoiding its use seems reasonable.

We also discovered that playfulness was positively and significantly related to the four lighter styles (i.e., Fun, Humor, Nonsense, and Wit), while no relationship was observed between playfulness and the darker styles, both in the whole group of participants and in the Whiteface and the Auguste samples separately. This seems reasonable, for previous research has highlighted that playfulness is related to adaptive humor, such as affiliative and self-enhancing humor, and is unrelated to aggressive and self-defeating humor [32,33]. Playfulness represents a personality trait that predicts individual differences in disposition to engage in play and the tendency to act spontaneously, pleasantly, creatively, voluntarily, and without objectives [19,34,35]. The tendency toward playfulness is related to happiness, positive impact, and relational satisfaction [36]. Moreover, lighter styles are generally related to positive effects and well-being [22,23]; therefore, it sounds reasonable that more playful clowns exhibit higher levels of adaptive humor. No difference emerged between the two characters (the Whiteface and the Auguste) in this regard. For both of them, playfulness was positively and significantly correlated to the four lighter styles. This result is in line with recent studies that have highlighted that clowns, independent of the role they play, tend to employ gentle play and positive humor during their activity [7,37,38].

The explained variance of the hierarchical regression model with the eight comic styles to predict playfulness styles was 27% with specific significance. Fun, Humor, Nonsense, and Wit positively predicted playfulness, while Cynicism was negatively linked to playfulness. When the two clown characters were considered separately, Fun and Humor were positively associated with playfulness in the Whiteface clowns, while the Auguste clowns showed only a positive association with Fun as a predictor of playfulness. As mentioned above, clowns are required to utilize gentle play, creativity, and empathy during their work, and the current study’s results reflect the importance of using positive humor to create a playful state of mind.

The results of this study highlight that the sample of clown doctors examined possessed both playfulness and adaptive humor. Significant differences were also observed concerning the different comic styles used by different characters. Although these findings shed light on a topic that, to date, has been only theoretically presented, some limitations to extending the findings to other clown doctors need to be acknowledged. First, as the study was conducted on Italian clown doctors, further research on medical clowns from different cultures and nationalities is necessary to confirm these results. Second, this sample primarily comprised volunteer clowns. Future studies on professional clowns should be conducted to identify any possible differences. The third limitation concerns the correlational nature of the research; therefore, future research should investigate the causal processes that link the considered variables. Finally, future studies should be conducted in order to examine the fine-grained facets of playfulness that allow derivation of information on what types of playfulness might be fruitful for clown doctors. Since the state-of-the-art model distinguishes between four types of playfulness [39], it is reasonable to expect that the facets of Other-directed playfulness and Intellectual playfulness in particular would be helpful to reframe situations in a way that they are experienced as interpersonally comforting and interesting [40].

In conclusion, this is the first study that focuses on playfulness and different forms of humor used by clown doctors, demonstrating specific differences between the two roles played and with the general population. Wide-ranging practitioners in this field, such as clowns, trainers, and researchers, may gain knowledge from these results. Playfulness represents a positive trait of clown doctors that can influence the way they utilize humor and vice versa, taking into account the personal characteristics and experiences of the clowns. This represents a relevant aspect, as people who approach the art of clowning in healthcare settings are required to undergo both psychological and artistic training focused on working with playfulness at the initial level and also throughout the training to enhance their ability to employ positive forms of humor. Moreover, as people tend to play different roles when acting as clown doctors, being aware of the specific kinds of humor used by the two characters may facilitate the initial and ongoing training they undergo in order to provide a higher quality of the course and of the activity. This study furnishes this assumption with a scientific background and provides empirical evidence that clowns must undergo ongoing training aimed at fostering these two aspects.

## Figures and Tables

**Table 1:** Mean scores and standard deviations (in parentheses) of Experience/Characters and CSM/SMAP.

	<1–4 Years M (SD)		4–9 Years M (SD)		>9 Years M (SD)		F (5, 210)	η^2^
	W	A	W	A	W	A		
*SMAP*								
Playfulness	5.7 (0.68)	6.0 (0.62)	5.7 (0.63)	6.0 (0.69)	5.8 (0.56)	5.6 (0.91)	0.68	0.01
*CSM*								
Fun	5.1 (1.06)	4.8 (1.21)	4.9 (1.08)	5.1 (0.94)	4.9 (1.09)	4.4 (1.08)	2.01	0.02
Humor	5.2 (0.78)	5.3 (0.85)	5.4 (0.70)	5.2 (0.64)	5.3 (0.90)	5.3 (0.74)	0.10	0.00
Nonsense	5.2 (1.05)	5.2 (0.96)	5.3 (1.05)	5.1 (0.91)	5.1 (1.13)	5.2 (0.74)	0.00	0.00
Wit	4.9 (0.83)	4.6 (1.14)	4.8 (0.94)	4.9 (0.95)	5.0 (0.77)	4.6 (0.86)	0.17	0.00
Irony	**4.4 ^ab^ (1.22)**	**4.1 ^ab^ (1.23)**	**4.6 ^b^ (1.09)**	**4.3 ^b^ (1.09)**	**4.1 ^a^ (1.30)**	**3.7 ^a^ (1.03)**	3.39 *	0.03
Satire	4.4 (1.24)	4.3 (1.17)	4.5 (0.99)	4.2 (1.05)	4.0 (1.40)	4.0 (1.02)	2.01	0.02
Sarcasm	**3.4 ^ab^ (1.20)**	**3.4 ^ab^ (1.08)**	**3.8 ^b^ (1.07)**	**3.4 ^b^ (1.01)**	**3.4 ^a^ (1.36)**	**2.7 ^a^ (1.11)**	3.38 *	0.03
Cynicism	**3.7 ^ab^ (1.30)**	**3.5 ^ab^ (1.11)**	**3.9 ^b^ (1.17)**	**3.6 ^b^ (0.73)**	**3.5 ^a^ (1.22)**	**3.0 ^a^ (1.06)**	3.12 *	0.03

Note: M = Mean; SD = Standard Deviation; W = Whiteface Clown; A = Auguste Clown. * *p* > 0.05. Tukey a, b for experience and characters. Significant differences are noted in **bold**.

**Table 2:** Mean scores (and standard deviations in parentheses) of Clowns and Non-clowns and CSM.

	M (SD) CLOWNS	M (SD) NON-CLOWNS	*F* (1, 817)	η^2^
*N*	210 (25.7%)	607 (74.3%)		
*CSM*				
Fun	**4.9 (1.09)**	**4.2 (1.29)**	44.88 ***	0.05
Humor	**5.3 (1.09)**	**5.1 (0.98)**	8.45 **	0.01
Nonsense	**5.2 (0.96)**	**4.7 (1.31)**	17.06 ***	0.02
Wit	4.8 (0.95)	4.8 (1.08)	0.27	0.00
Irony	4.2 (1.17)	4.3 (1.16)	1.63	0.00
Satire	4.2 (1.14)	4.4 (1.18)	2.44	0.00
Sarcasm	3.3 (1.15)	3.5 (1.17)	2.65	0.00
Cynicism	**3.5 (1.14)**	**3.8 (1.28)**	7.60 *	0.00

Note: data of non-clowns come from the Italian Validation of Comic Styles Markers [23]. *** *p* > 0.001. ** *p* > 0.01. * *p* > 0.05. Significant differences are noted in **bold**.

**Table 3:** Standardized betas and proportion explained variance for the regression analyses of SMAP on CSM as predictors in the whole sample and the Whiteface and the Auguste clowns (correlation in parenthesis).

	Total (SMAP)	White	August
*CSM*			
Fun	0.41 *** (0.363 *** **)**	0.39 ** (0.321 *** **)**	0.38 ** (0.402 *** **)**
Humor	0.22 *** (.335 *** **)**	0.35 ** (0.320 *** **)**	0.18 (0.350 ***)
Nonsense	0.08 ** (0.319 *** **)**	−0.00 (0.238 **)	0.16 (0.391 ***)
Wit	0.10 (0.290 ***)	−0.02 (0.213 *)	0.19 (0.350 ***)
Irony	−0.06 (0.106)	0.04 (0.093)	−0.06 (0.137)
Satire	−0.13 (0.093)	−0.34 (−0.036)	−0.12 (0.190 *)
Sarcasm	−0.00 (0.039)	0.05 (0.006)	−0.05 (0.084)
Cynicism	−0.20 * (−0.029)	−0.09 (−0.023)	−0.18 (−0.011)
*R^2^*	0.27 ***	0.25 **	0.31 ***

*** *p* < 0.001. ** *p* < 0.01. * *p* < 0.05. Significant differences are noted in **bold**.

## Data Availability

The datasets generated and/or analyzed during the current study are available from the corresponding author upon reasonable request.

## References

[1] Finlay F., Baverstock A., Lenton S. (2014). Therapeutic clowning in paediatric practice. Clin. Child Psychol..

[2] Warren B., Spitzer P. (2013). Smiles Are Everywhere: Integrating Clown-Play into Healthcare Practice.

[3] Lopes-Júnior L.C., Bomfim E., Olson K., Neves E.T., Silveira D.S.C., Nunes M.D.R., Lima R.A.G. (2020). Effectiveness of hospital clowns for symptom management in paediatrics: Systematic review of randomised and non-randomised controlled trials. BMJ.

[4] Sridharan K., Sivaramakrishnan G. (2016). Therapeutic clowns in pediatrics: A systematic review and meta-analysis of randomized controlled trials. Eur. J. Pediatr..

[5] Mac Farlane V.V. (2022). Integration of healthcare clowns into pediatric palliative care: A bridge between life and death. IJIC.

[6] Blain S., Kingsnorth S., Stephens L., McKeever P. (2012). Determining the effects of therapeutic clowning on nurses in a children's rehabilitation hospital. Arts Health.

[7] Holland M., Fiorito M.E., Gravel M.L., McLeod S., Polson J., Incio Serra N., Blain-Moraes S. (2022). “We are still doing some magic”: Exploring the effectiveness of online therapeutic clowning. Arts Health.

[8] Peacock L. (2009). Serious Play: Modern Clown Performance.

[9] Koller D., Gryski C. (2008). The life threatened child and the life enhancing clown: Towards a model of therapeutic clowning. eCAM.

[10] Dionigi A. (2016). Personality of clown doctors: An exploratory study. J. Individ. Differ..

[11] Dionigi A., Ruch W., Platt T. (2014). Components and determinants of the shift between own persona and the clown persona: A hierarchical analysis. Eur. J. Humour Res..

[12] Dionigi A., Casu G., Gremigni P. (2020). Associations of self-efficacy, optimism, and empathy with psychological health in healthcare volunteers. Int. J. Environ. Res. Public Health.

[13] Vagnoli L., Brauer K., Addarii F., Ruch W., Marangi V. (2022). Fear of being laughed at in Italian healthcare workers: Testing associations with humor styles and humor coping. Curr. Psychol..

[14] Linge L. (2008). Hospital clowns working in pairs—In synchronized communication with ailing children. Int. J. Qual. Stud. Health Well-Being..

[15] Linge L. (2012). Magical attachment: Children in magical relations with hospital clowns. Int. J. Qual. Stud Health Well-Being.

[16] Nuttman-Shwartz O., Scheyer R., Tzioni H. (2010). Medical clowning: Even adults deserve a dream. Soc. Work. Health Care.

[17] McGhee P.E. (1996). Health, Healing and the Amuse System: Humor as Survival Training.

[18] Proyer R.T. (2018). Playfulness and humor in psychology: An overview and update. Humor.

[19] Proyer R.T., Tandler N., Brauer K., Luria S.R., Baer J., Kaufman J.C. (2019). Playfulness and creativity: A selective review. Creativity and Humor.

[20] Martin R.A., Ford T. (2018). The Psychology of Humor: An Integrative Approach.

[21] Martin R.A., Puhlik-Doris P., Larsen G., Gray J., Weir K. (2003). Individual differences in uses of humor and their relation to psychological well-being: Development of the humor styles questionnaire. J. Res. Pers..

[22] Ruch W., Heintz S., Platt T., Wagner L., Proyer R.T. (2018). Broadening humor: Comic styles differentially tap into temperament, character, and ability. Front. Psychol..

[23] Dionigi A., Duradoni M., Vagnoli L. (2021). Humor and anxiety: The relationship between the comic styles, worry and general well-being. Pers. Individ. Differ..

[24] Proyer R.T. (2012). Development and initial assessment of a short measure for adult playfulness: The SMAP. Pers. Individ. Differ..

[25] Ruch W., Wagner L., Heintz S. (2018). Humor, the PEN model of personality, and subjective well-being: Support for differential relationships of eight comic styles. RISU.

[26] Ford K., Courtney-Pratt H., Tesch L., Johnson C. (2014). More than just clowns–Clown doctor rounds and their impact for children, families and staff. J. Child. Health Care.

[27] Auerbach S., Ruch W., Fehling A. (2016). Positive emotions elicited by clowns and nurses: An experimental study in a hospital setting. Transl. Issues Psychol..

[28] Dowling J.S. (2002). Humor: A coping strategy for. Pediatr. Nurs..

[29] Heintz S., Ruch W. (2019). From four to nine styles: An update on individual differences in humor. Pers. Individ. Differ..

[30] Ruch W., McGhee P.E., Hehl F.J. (1990). Age differences in the enjoyment of incongruity-resolution and nonsense humor during adulthood. Psychol. Aging.

[31] McGhee P.E., Frank M. (2014). Humor and Children’s Development: A Guide to Practical Applications.

[32] Ruch W.F., Heintz S. (2013). Humour styles, personality and psychological well-being: What’s humour got to do with it?. Eur. J. Humour Res..

[33] Yue X.D., Leung C.L., Hiranandani N.A. (2016). Adult playfulness, humor styles, and subjective happiness. Psychol. Rep..

[34] Barnett L.A. (1991). The playful child: Measurement of a disposition to play. Play Cult..

[35] Proyer R.T., Gander F., Brauer K., Chick G. (2021). Can playfulness be stimulated? A randomised placebo-controlled online playfulness intervention study on effects on trait playfulness, well-being, and depression. Appl. Psychol. Health Well-Being.

[36] Proyer R.T., Gander F., Wellenzohn S., Ruch W. (2014). Positive psychology interventions in people aged 50–79 years: Long-term effects of placebo-controlled online interventions on wellbeing and depression. Aging Ment. Health.

[37] Karnieli-Miller O., Divon-Ophir O., Sagi D., Pessach-Gelblum L., Ziv A., Rozental L. (2023). More than just an entertainment show: Identification of medical clowns’ communication skills and therapeutic goals. Qual. Health Res..

[38] Warren B., Twohig P., Kalitzkus V. (2004). Treating wellness: How clown doctors help to humanize health care and promote good health. Making Sense of: Health, Illness and Disease.

[39] Proyer R.T. (2017). A new structural model for the study of adult playfulness: Assessment and exploration of an understudied individual differences variable. Personal. Individ. Differ..

[40] Brauer K., Proyer R.T., Chick G. (2021). Adult playfulness: An update on an understudied individual differences variable and its role in romantic life. Soc. Personal. Psychol. Compass.

